# Neurotrophic Effect of Citrus 5-Hydroxy-3,6,7,8,3′,4′-Hexamethoxyflavone: Promotion of Neurite Outgrowth via cAMP/PKA/CREB Pathway in PC12 Cells

**DOI:** 10.1371/journal.pone.0028280

**Published:** 2011-11-29

**Authors:** Hui-Chi Lai, Ming-Jiuan Wu, Pei-Yi Chen, Ting-Ting Sheu, Szu-Ping Chiu, Meng-Han Lin, Chi-Tang Ho, Jui-Hung Yen

**Affiliations:** 1 Department of Molecular Biology and Human Genetics, Tzu Chi University, Hualien, Taiwan; 2 Department of Biotechnology, Chia-Nan University of Pharmacy and Science, Tainan, Taiwan; 3 Institute of Medical Science, Tzu Chi University, Hualien, Taiwan; 4 Center of Medical Genetics, Buddhist Tzu Chi General Hospital, Hualien, Taiwan; 5 Institute of Microbiology, Immunology and Biochemistry, Tzu Chi University, Hualien, Taiwan; 6 Department of Food Science, Rutgers University, New Brunswick, New Jersey, United States of America; University of Pittsburgh, United States of America

## Abstract

5-Hydroxy-3,6,7,8,3′,4′-hexamethoxyflavone (5-OH-HxMF), a hydroxylated polymethoxyflavone, is found exclusively in the Citrus genus, particularly in the peels of sweet orange. In this research, we report the first investigation of the neurotrophic effects and mechanism of 5-OH-HxMF in PC12 pheochromocytoma cells. We found that 5-OH-HxMF can effectively induce PC12 neurite outgrowth accompanied with the expression of neuronal differentiation marker protein growth-associated protein-43(GAP-43). 5-OH-HxMF caused the enhancement of cyclic AMP response element binding protein (CREB) phosphorylation, c-fos gene expression and CRE-mediated transcription, which was inhibited by 2-naphthol AS-E phosphate (KG-501), a specific antagonist for the CREB-CBP complex formation. Moreover, 5-OH-HxMF-induced both CRE transcription activity and neurite outgrowth were inhibited by adenylate cyclase and protein kinase A (PKA) inhibitor, but not MEK1/2, protein kinase C (PKC), phosphatidylinositol 3-kinase (PI3K) or calcium/calmodulin-dependent protein kinase (CaMK) inhibitor. Consistently, 5-OH-HxMF treatment increased the intracellular cAMP level and downstream component, PKA activity. We also found that addition of K252a, a TrKA antagonist, significantly inhibited NGF- but not 5-OH-HxMF-induced neurite outgrowth. These results reveal for the first time that 5-OH-HxMF is an effective neurotrophic agent and its effect is mainly through a cAMP/PKA-dependent, but TrKA-independent, signaling pathway coupling with CRE-mediated gene transcription. A PKC-dependent and CREB-independent pathway was also involved in its neurotrophic action.

## Introduction

Neurotrophic factors, such as nerve growth factor (NGF) and brain-derived neurotrophic factor (BDNF) have been reported to promote neurogenesis, neural development, neuronal survival and functional maintenance of neurons [1], [2]. The levels of neurotrophic factors are changed in a wide variety of neurodegenerative disorders, such as Alzheimer's disease (AD), Parkinson's disease, Huntington's disease and amyotrophic lateral sclerosis [3]. NGF is known as the most efficacious neurotrophic factor acting on the basal forebrain cholinergic neurons (BFCNs) and suggested as a potential therapeutic agent for degeneration of cholinergic neurons in patients with AD [2]. However, NGF is a large size of polypeptide and its supplementation on the peripheral administration is severely restricted by the difficulties in the blood-brain-barrier (BBB) penetration [4]. In addition, NGF has been reported to interact at low affinity with p75 receptor and lead to undesirable effects such as pain [5]. As a result, identification of small molecules that can mimic the neuritogenic ability of NGF and substitute for its clinical use serves as an alternative therapy approach [6].

PC12 pheochromocytoma cell line is a widely used model system for studies of neuronal cell differentiation, neuronal survival, and neurotransmitter secretion, as well as defining the underlying molecular mechanisms [7]. Exposure of PC12 cells to NGF triggers differentiation into sympathetic-like neuronal cells, characterized by long-term and stable neurite outgrowth and exhibiting many properties of adrenal medullary chromaffin cells, including catecholamine synthesis, storage, and secretion [8]. NGF induces rapid tyrosine phosphorylation of trkA and consequent phosphorylation and activation of signal transduction substrates including extracellular signal regulated kinases (ERKs)/mitogen-activated protein kinases (MAPKs) [9], [10]. It has been reported that NGF-mediated ERK activation induces phosphorylation of cAMP-response element binding protein (CREB), which further recruits the CREB binding protein (CBP) to the promoter regions of cAMP-responsive genes associated with dendritic spine growth, morphology change, synaptic plasticity, and long-term memory [11], [12]. In addition to ERK/MAPK, various individual signaling cascades may converge signal to the CREB, including protein kinase C (PKC) [13], cAMP-dependent protein kinase A (PKA) [14], [15], phosphatidylinositol 3-kinase (PI3K)/Akt [16], [17], and calcium/calmodulin-dependent protein kinase (CaMK) [18], [19].

**Figure 1:**
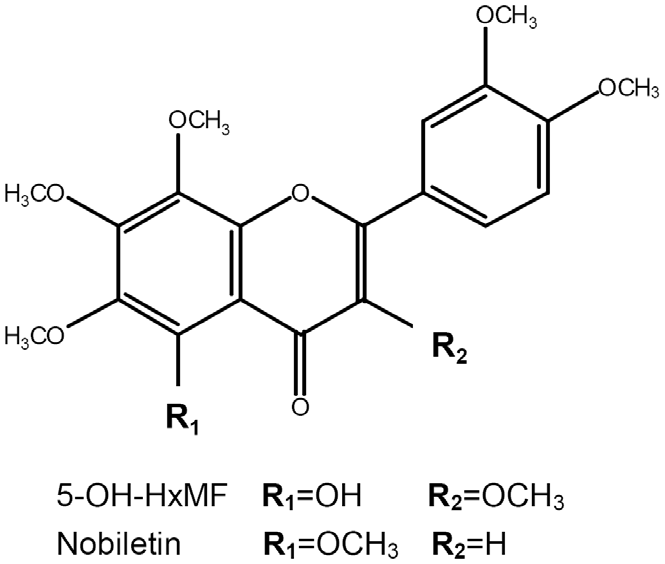
Chemical structure of 5-Hydroxy-3,6,7,8,3′,4′-hexamethoxyflavone (5-OH-HxMF) and nobiletin.

It is known that several phytochemicals derived from daily consumed vegetables and fruits are associated with disease-preventing effects. Flavonoids, a family of polyphenolic phytochemicals, have been shown to alter cell signaling and gene expression which might contribute to their purported physiological benefits [20], [21]. Polymethoxyflavones (PMFs) exist almost exclusively in the peels of citrus such as sweet orange (*Citrus sinensis* (L.) Osbeck) and mandarin orange (*Citrus reticulate* Blanco). The roles of PMFs in prevention and treatment of diseases have received considerable attention recently, with particular interest in the use of these citrus flavonoids as antioxidant, anti-inflammatory, anti-cancer, and anti-atherogenic agents [22]–[26]. Nobiletin (**Figure 1**), the most abundant and studied PMF in orange peel extract [23], has been found to induce neurite outgrowth through cAMP and ERK/MAPK-dependent mechanism [27], to stimulate CRE transcription activity [28], to enhance long-term potentiation (LTP) in the hippocampal slices [29], and to improve impaired memory in animal models including olfactory-bulbectomized, Alzheimer's disease, and brain-ischemia mice [30]–[32]. A metabolite of nobiletin, 4′-demethylnobiletin, has also been reported to rescue learning impairment via stimulation of ERK and CREB signaling pathways [33]. The recent isolation of 5-hydroxy-3,6,7,8, 3′,4′-hexamethoxyflavone (5-OH-HxMF) (**Figure 1**), a 5-hydroxylated PMF, from sweet orange peel extract [23] and the reported biological activities of relevant polymethoxyflavones mentioned above promoted us to study its neurotrophic and neuroprotective activities. In the present study, we focused on the neurotrophic effect of 5-OH-HxMF on promoting neurite outgrowth and neuronal differentiation in PC12 cells. We also explored the possible signaling molecular pathways associated with its neurotrophic action. It is noteworthy that this is the first report on the neurotrophic action and mechanism of 5-hydroxylated PMFs.

## Results

### 5-OH-HxMF promotes PC12 neurite outgrowth

PC12 cell line is widely used as a cellular model for studies of neurotrophic action [8]. To evaluate the effect of the 5-hydroxylated polymethoxyflavone on cell viability in PC12 cell system, cells were maintained in the low serum medium (1% HS and 0.5% FBS) and treated with indicated concentration of vehicle (0.1% DMSO), NGF (50 ng/ml = 0.38 nM; as a positive control), 5-OH-HxMF or nobiletin (0–50 µM) for 48 h. The relative cell counts were analyzed using MTT assay and values were expressed as percentage of control group. As shown in **Figure S1**, NGF exerted a proliferative effect in PC12 cells in low serum culture condition. The 5-OH-HxMF and nobiletin showed no further decrease in viability and sustained PC12 cell survival in low serum medium. This result reveals none of these two PMF compounds exhibited cytotoxicity for 48 h incubation in this low serum culture system. To evaluate the neuritogenic action in our cell system, adherent PC12 cells in low serum medium (1% HS and 0.5% FBS) were treated with neurotrophic factor NGF (25–100 ng/ml = 0.19–0.76 nM) for quantification of neurite outgrowth. Cell morphology was observed and percentage of neurite-bearing cells was counted by phase contrast microscopy. **Figure S2** shows that NGF significantly promoted neurite outgrowth of low-serum cultured PC12 cells in a dose- and time-dependent manner. Growth-associated protein-43 (GAP-43) is a neuron-specific protein which exhibits increased synthesis and axonal fast-transport during nerve regeneration. The correlation of elevated GAP-43 expression with neuronal growth states is well established [34], [35]. To confirm our morphological observation at the molecular level, we immunostained for GAP-43 expression after NGF (100 ng/ml = 0.76 nM) treatment for 48 h. NGF-differentiated PC12 cells displayed extensive expression of the neuronal differentiation marker GAP-43 (**Figure S2C**).

**Figure 2:**
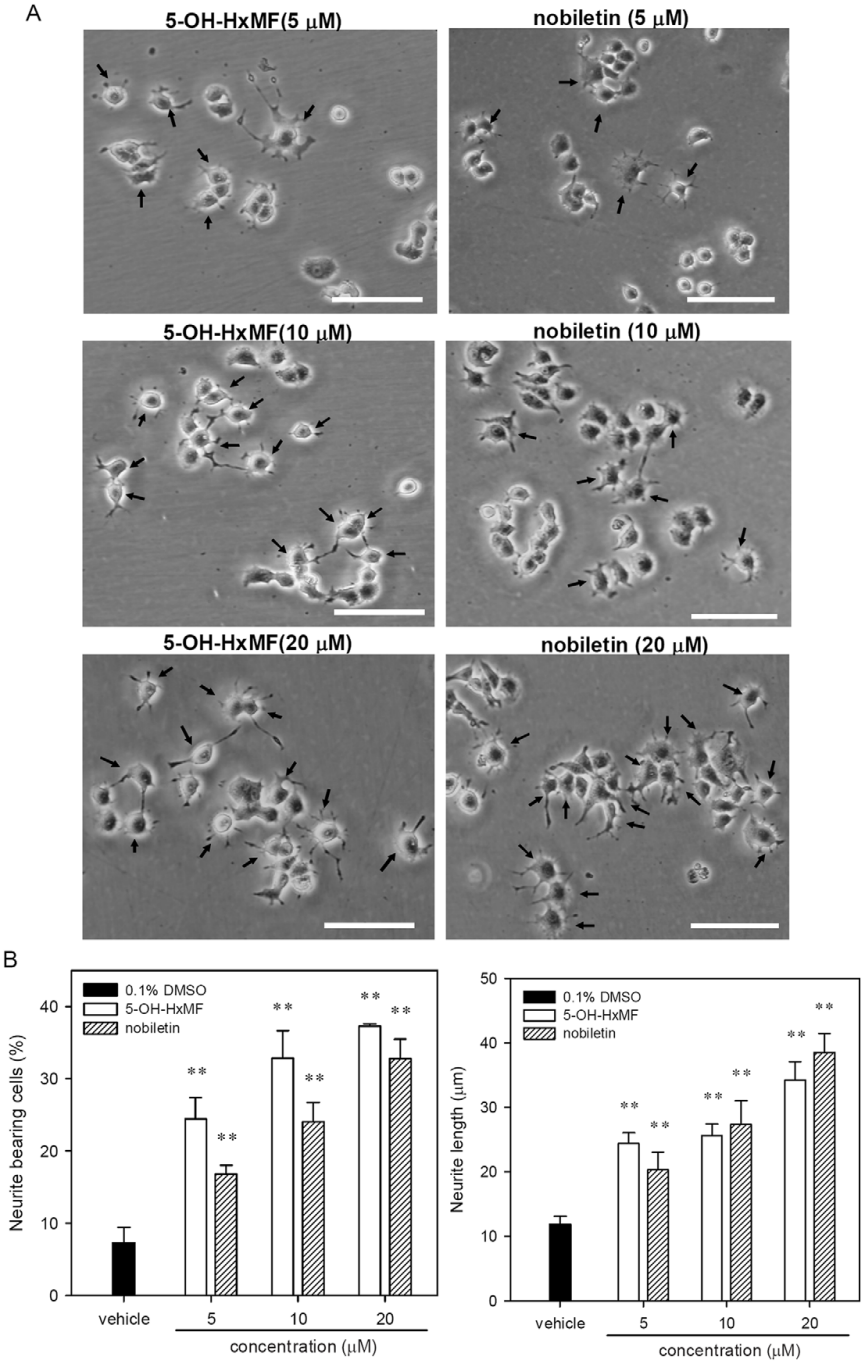
Morphological characteristics of 5-OH-HxMF- and nobiletin-treated PC12 cells. PC12 cells were seeded on poly-L-lysine-coated 6-well plates in low serum medium for 24 h prior to exposure to vehicle (0.1% DMSO) or indicated agents for additional 48 h. Cell morphology was observed using phase-contrast microscopy and photographed by the digital camera. Arrowheads indicate the neurite bearing cells in 5-OH-HxMF- or nobiletin-treated groups. Scale bar, 100 µm. (**B**) Neurite bearing cells were analyzed as described in Materials and Methods. Data represent the mean ± SD from three independent experiments. The maximal neurite length for each of the differentiated cells was analyzed and the average neurite length was calculated as described in Materials and Methods. Data represent the mean ± SD from three independent experiments. **p<0.01 represents significant differences compared with those of the vehicle-treated cells.

To investigate whether citrus polymethoxyflavones also induce neurite outgrowth in PC12 cells, adherent cells maintained in low serum medium were treated with vehicle (0.1% DMSO), 5-OH-HxMF, or nobiletin (5, 10, and 20 µM) for 48 h for analysis of cell morphology change (**Figure 2A**). As shown in **Figure 2B**, quantification of neurite outgrowth data showed that treatment of PC12 cells with 5-OH-HxMF and nobiletin significantly evoked a dose-dependent increase on neurite outgrowth and the percentage of neurite-bearing cells markedly reached 24.4±3.0%, 32.8±2.7%, and 37.3±0.3% for 5 µM, 10 µM, and 20 µM 5-OH-HxMF; 16.8±1.2%, 24.0±2.7% and 32.9±3.8% for 5 µM, 10 µM, and 20 µM nobiletin, respectively (*p<*0.01). 5-OH-HxMF and nobiletin also significantly increased the maximal neurite length in those cells (24.4±1.7 µm, 25.7±1.8 µm, and 34.3±2.8 µm for 5 µM, 10 µM, and 20 µM 5-OH-HxMF; 20.3±2.8 µm, 27.4±3.6 µm, and 38.5±3.0 µm for 5 µM, 10 µM, and 20 µM nobiletin, respectively) as compared with those of the vehicle control (11.9±1.2 µm) (*p<*0.01). These above results indicate that 5-OH-HxMF and nobiletin possess compatible neurotrophic activity as NGF.

### 5-OH-HxMF promotes GAP-43 expression in PC12 cells

**Figure 3:**
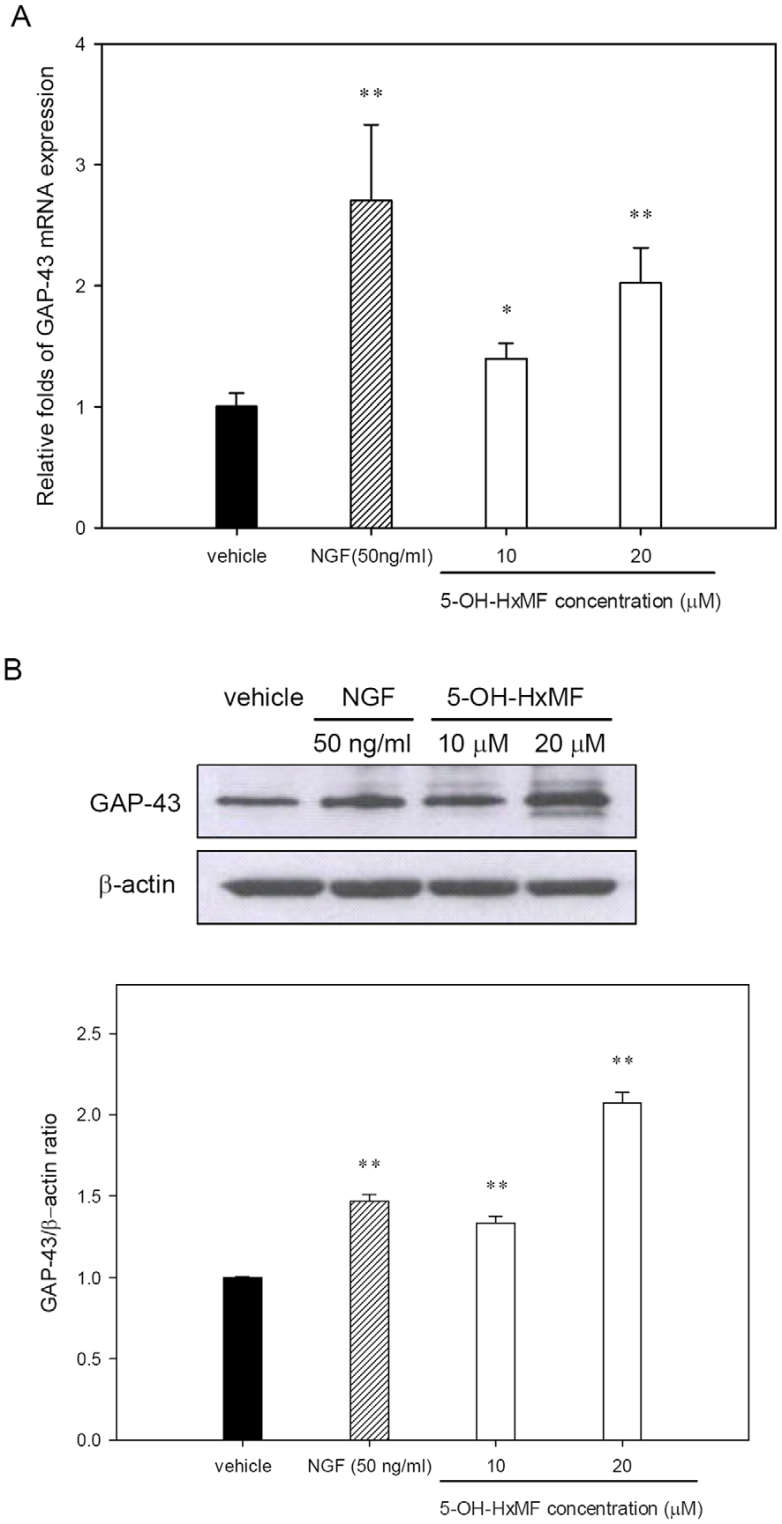
Effects of 5-OH-HxMF on GAP-43 mRNA and protein expression in PC12 cells. PC12 cells were seeded on poly-L-lysine-coated 6-well plates or 100 mm dishes in normal medium for 24 h and then shifted to low serum medium (1% HS and 0.5% FBS) for 24 h prior to exposure to indicated agents. **(A)** Cells were treated with vehicle (0.1% DMSO), NGF (50 ng/ml; 0.38 nM) and 5-OH-HxMF (10 and 20 µM) for 24 h, respectively. Cellular RNA was then prepared and GAP-43 mRNA level was detected by RT-Q-PCR as described in Materials and Methods. Data represent the mean ± SD of three independent experiments. **(B)** Cells were treated with vehicle (0.1% DMSO), NGF (50 ng/ml; 0.38 nM) and 5-OH-HxMF (10 and 20 µM) for 24 h. GAP-43 protein expression was detected by Western blotting as described in Materials and Methods. The immunoblot experiments were replicated three times and a representative blot was shown. Normalized intensity of GAP-43 versus β-actin is presented as the mean ± SD of three independent experiments. *p<0.05 and **p<0.01 represent significant differences compared with vehicle-treated cells.

To further confirm the neurotrophic effect of 5-OH-HxMF, GAP-43 expression was determined by Western blot analysis and RT-Q-PCR. **Figure 3A** shows NGF (50 ng/ml = 0.38 nM) significantly increased GAP-43 mRNA expression (approximately 2.5-fold) over vehicle control (*p*<0.01). Cells treated with 10 and 20 µM 5-OH-HxMF for 24 h caused 1.4- and 2.0-fold increases in the GAP-43 transcripts, respectively. Western blot analysis reveals GAP-43 protein expression was significantly induced by 5-OH-HxMF after 24 h of treatment. GAP-43 protein levels were elevated 1.5-, 1.3-, and 2.1-fold in response to NGF (50 ng/mL = 0.38 nM), 10 and 20 µM 5-OH-HxMF, respectively (**Figure 3B**). These results reveal that 5-OH-HxMF induces the mRNA and protein expression of GAP-43 associated with the differentiation of PC12 cells into neuronal phenotype.

### 5-OH-HxMF stimulates phosphorylation of CREB in PC12 cells

**Figure 4:**
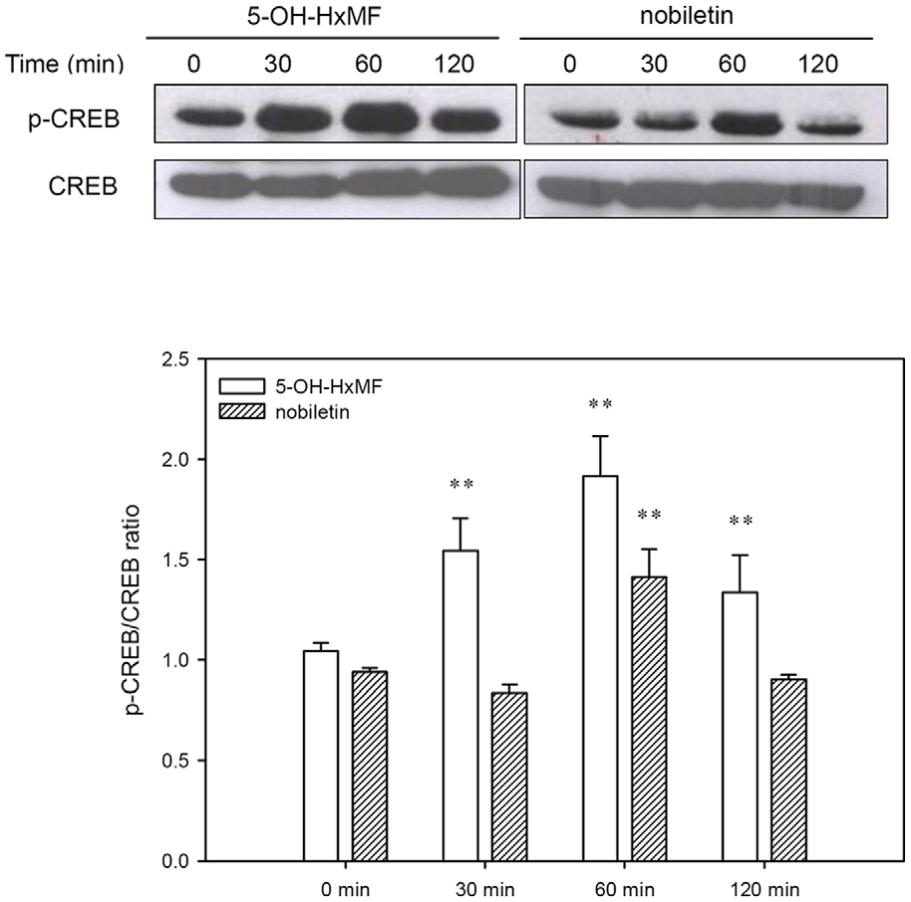
Effects of 5-OH-HxMF and nobiletin on the phosphorylation of CREB protein in PC12 cells. PC12 cells were seeded on poly-L-lysine-coated 100 mm dishes in normal medium for 24 h and then shifted to low serum medium (1% HS and 0.5% FBS) for 24 h prior to exposure to indicated agents. Cells were treated with vehicle (0.1% DMSO), 5-OH-HxMF or nobiletin (20 µM) for 0 min, 30 min, 60 min or 120 min. Phosphor-CREB (p-CREB) and CREB proteins were analyzed by Western blotting as described in Materials and Methods. The immunoblot experiments were replicated at least three times and a representative blot was shown. Normalized intensity of p-CREB versus CREB is presented as the mean ± SD of three independent experiments. **p<0.01 represent significant differences compared with 0 min group.

Several reports suggested that phosphorylation of transcription factor CREB played critical roles for neurogenesis and neuronal differentiation [36]–[38]. To investigate whether 5-OH-HxMF can activate CREB signaling, PC12 cells were treated with 20 µM 5-OH-HxMF and nobiletin for indicated period, and cell lysate was immunoblotted with phospho-CREB (p-CREB) and CREB specific antibodies as described in Materials and Methods. As shown in **Figure 4**, treatment of PC12 cells with 20 µM 5-OH-HxMF increased CREB phosphorylation by 1.5-fold within 30 min, peaked by 60 min (2-fold), and lasted until 120 min (1.3-fold) as compared with 0 min group (*p*<0.01). In comparison, CREB phosphorylation peaked at 60 min by nobiletin and then rapidly decreased. This data shows that 5-OH-HxMF and nobiletin activate rapid and transient CREB phosphorylation in PC12 cells.

### Effects of 5-OH-HxMF-induced CREB phosphorylation on the CRE-dependent transcription activity and neurite outgrowth in PC12 cells

**Figure 5:**
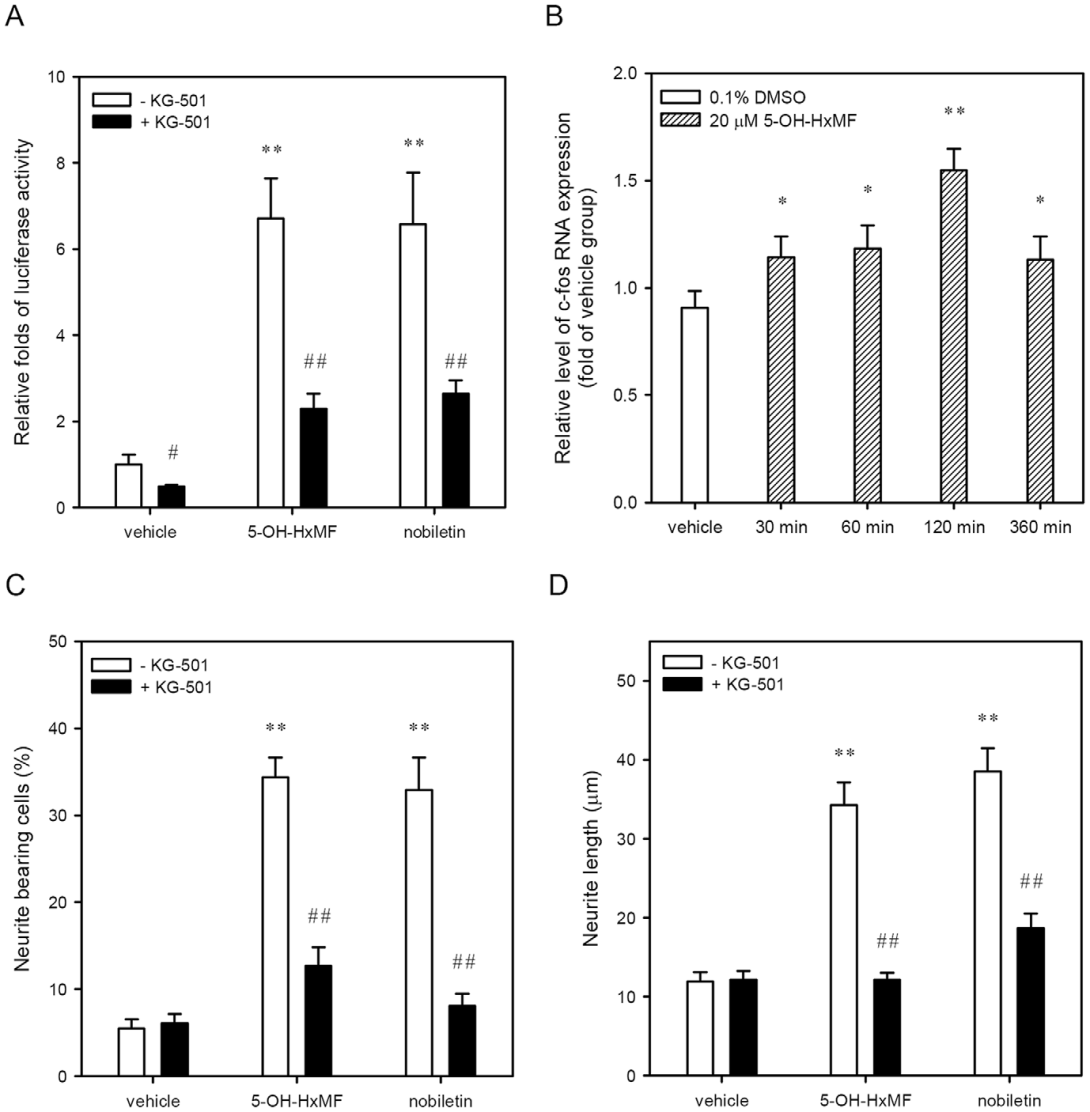
Effects of 5-OH-HxMF, nobiletin, and inhibitor KG-501 on CRE-dependent gene transcription and neurite outgrowth in PC12 cells. (**A**) PC12 cells were transfected with a CRE-mediated luciferase reporter construct and *Renilla* luciferase control plasmid for 24 h. After transfection, PC12 cells were treated with vehicle (0.1% DMSO), 5-OH-HxMF (20 µM), or nobiletin (20 µM) for 8 h. For treatment of cells with inhibitor, the transfected cells were pre-incubated with KG-501 (10 µM) for 30 min, then exposure of cells with vehicle, 5-OH-HxMF, or nobiletin for additional 8 h. Cells were harvested and luciferase activities were determined as described in Materials and Methods. The intensity of the luciferase reactions measured in the lysates of the transfectants was normalized to their *Renilla* luciferase control activity. (**B**) PC12 cells were cultured on poly-L-lysine-coated 6-well plates in low serum medium and treated with vehicle (0.1% DMSO) and 5-OH-HxMF (20 µM) for indicated period. Cellular RNA was then prepared and c-fos mRNA level was detected by RT-Q-PCR as described in Materials and Methods. Data represent the mean ± SD of three independent experiments. (**C**) PC12 cells were seeded on poly-L-lysine-coated 6-well plates in normal medium for 24 h and then shifted to low serum medium (1% HS and 0.5% FBS) for 24 h prior to exposure to vehicle (0.1% DMSO), 5-OH-HxMF (20 µM) or nobiletin (20 µM) for additional 48 h. For treatment of cells with inhibitor, adherent cells were pre-incubated with KG-501 (10 µM) for 30 min and then exposure to indicated agents for additional 48 h. Neurite bearing cells were analyzed as described in Materials and Methods. Data represent the mean ± SD from three independent experiments. (**D**) The maximal neurite length for each of the differentiated cells was analyzed and the average neurite length was calculated as described in Materials and Methods. Data represent the mean ± SD from three independent experiments. *p<0.05 and **p<0.01 represent significant differences compared with vehicle-treated cells. #*p*<0.05 and ##*p*<0.01 represent significant differences compared with respective KG-501-untreated group.

To determine whether 5-OH-HxMF-induced CREB phosphorylation can also activate the transcription activity of cAMP response element (CRE), the CRE-mediated luciferase reporter plasmid and *Renilla* internal control vector were co-transfected into the PC12 cells, then the luciferase activity was measured as described in Materials and Methods. As shown in **Figure 5A**, treatment of PC12 cells with 20 µM 5-OH-HxMF and nobiletin increased the luciferase activity by approximately 6.5-fold compared to vehicle control (*p*<0.01), respectively. The 5-OH-HxMF or nobiletin-mediated transcription activity was significantly reduced by treatment of cells with 10 µM 2-naphthol AS-E phosphate (KG-501), a specific antagonist which disrupts the CREB:CBP complex and attenuates target gene induction [39]. This result reveals that 5-OH-HxMF can induce transcription of CRE-dependent genes. In addition, we further determined the effect of 5-OH-HxMF on the CREB target gene, *c-fos*, in PC12 cells. As shown in **Figure 5B**, exposure of PC12 cells with 5-OH-HxMF (20 µM) increased *c-fos* mRNA expression within 30 min and peaked by 120 min. This result confirms 5-OH-HxMF can activate CREB, which in turn up-regulates downstream target gene- *c-fos*.

As shown in **Figure 5C**, KG-501 treatment for 48 h in culture medium also significantly attenuated the percentage of 5-OH-HxMF and nobiletin-induced neurite-bearing cells from 34.4±2.3% to 12.7±2.1% and 32.9±3.7% to 8.1±1.4%, respectively (*p*<0.01). The average maximal neurite length in those differentiated cells was also reduced to the vehicle control level (**Figure 5D**). These results imply that both 5-OH-HxMF and nobiletin upregulate neurite outgrowth through CREB activation.

### 5-OH-HxMF promotes CREB activation and neurite outgrowth through cAMP-dependent PKA signaling pathway in PC12 cells

**Figure 6:**
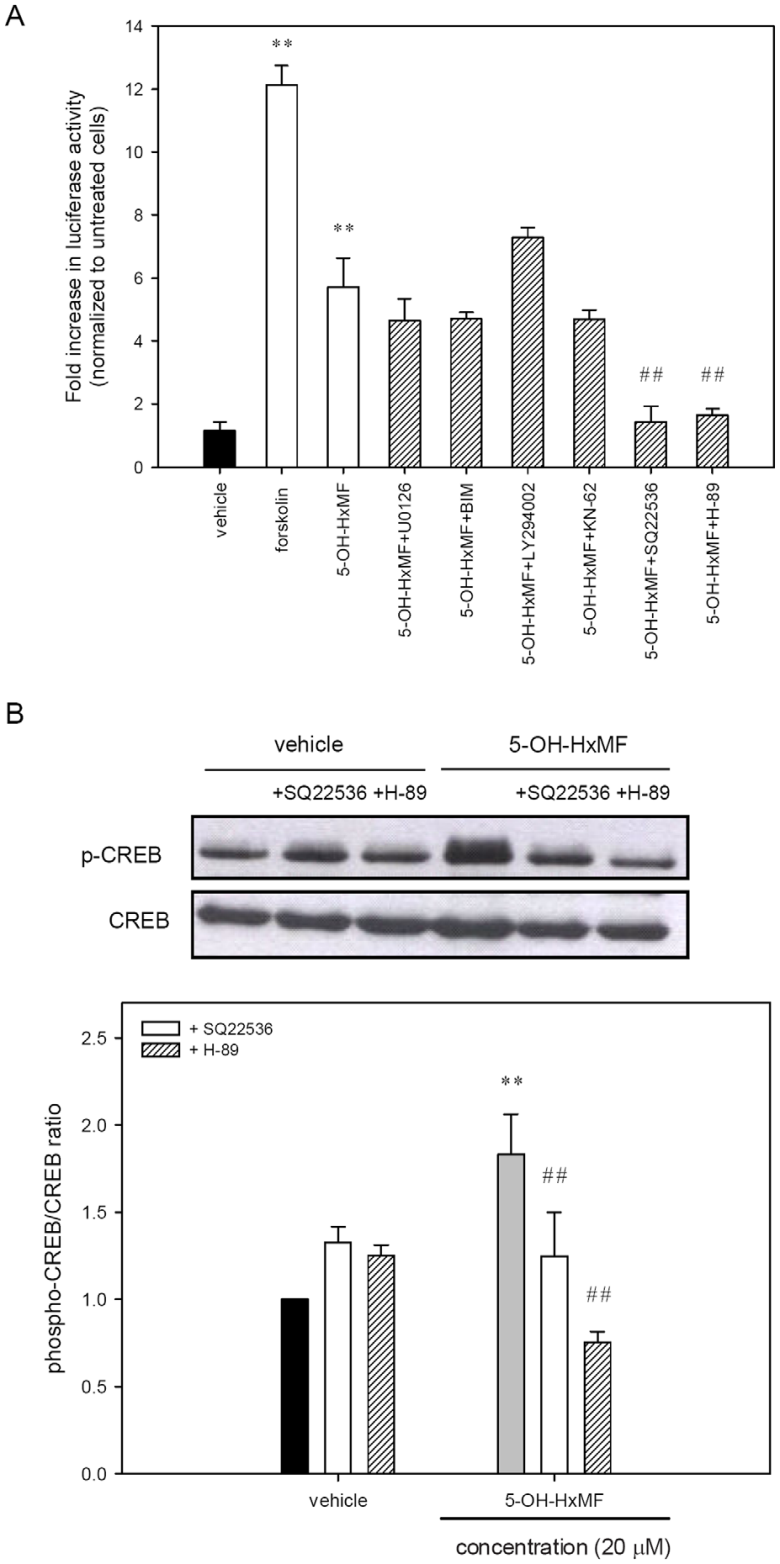
Effects of the molecular inhibitors on the 5-OH-HxMF -mediated CREB activation. (**A**) PC12 cells were transfected with a CRE-mediated luciferase reporter construct and *Renilla* luciferase control plasmid for 24 h. Following transfection, cells were pre-treated for 30 min with inhibitors 10 µM U0126, 2.5 µM BIM, 20 µM LY294002, 10 µM KN-62, 500 µM SQ22536, 10 µM H-89 or vehicle (0.1% DMSO), followed by exposure to 5-OH-HxMF (20 µM) for 8 h. Forskolin (2 µM)-treated transfected cells as a positive control for CRE-luciferase reporter assay. The intensity of the luciferase reactions measured in the lysates of the transfectants was normalized to their *Renilla* luciferase control activity. (**B**) PC12 cells were seeded on poly-L-lysine-coated 100 mm dishes in normal medium for 24 h and then shifted to low serum medium (1% HS and 0.5% FBS) for further 24 h culture. Cells were treated with inhibitor SQ22536 or H-89 for 30 min prior to exposure of vehicle (0.1% DMSO) or 5-OH-HxMF (20 µM) for 60 min. Phosphor-CREB (p-CREB) and CREB were analyzed by Western blotting as described in Materials and Methods. The immunoblot experiments were replicated at least three times and a representative blot was shown. Normalized intensity of p-CREB versus CREB is presented as the mean ± SD of three independent experiments. ** *p*<0.01 represents significant differences compared with vehicle. ## *p*<0.01 represents significant differences compared with respective inhibitors-untreated group.

Many data supported that CREB transcriptional regulation represents a crossing point for several signaling pathways and is known to regulate a variety of genes in neuronal functions [40]–[42]. To unravel the enigma, the possible involvement of ERK, PKC, PI3K/Akt, CaMK and cAMP-dependent PKA signaling in 5-OH-HxMF-upregulated CREB transcription activation was investigated by utilizing their molecular inhibitors. PC12 cells were transfected with reporter plasmids and treated with kinases specific inhibitors including MEK1/2 inhibitor (U0126; 10 µM), PKC inhibitor (bisindolylmaleimide I, BIM; 2.5 µM), PI3K inhibitor (LY294002; 20 µM), CaMK II inhibitor (KN-62; 10 µM), adenylate cyclase inhibitor (SQ22536; 500 µM) and PKA inhibitor (H-89; 10 µM) for 30 min, then incubated with 20 µM 5-OH-HxMF before analyzing the luciferase activity of cells. As shown in **Figure 6A**, 5-OH-HxMF as well as forskolin, an adenylate cyclase activator for CREB-mediated transcriptional activation, significantly increased the CRE-dependent transcription, as expected. Moreover, 5-OH-HxMF-mediated CREB-transcription activity was markedly attenuated by SQ22536 and H-89 to the vehicle control level (*p*<0.01). As shown in **Figure 6B** **,** immunoblot shows that SQ22536 and H-89 treatment of PC12 cells specifically abolished 5-OH-HxMF-mediated CREB phosphorylation by 32% and 60%, respectively (*p*<0.01), without changing those of vehicle control.

**Figure 7:**
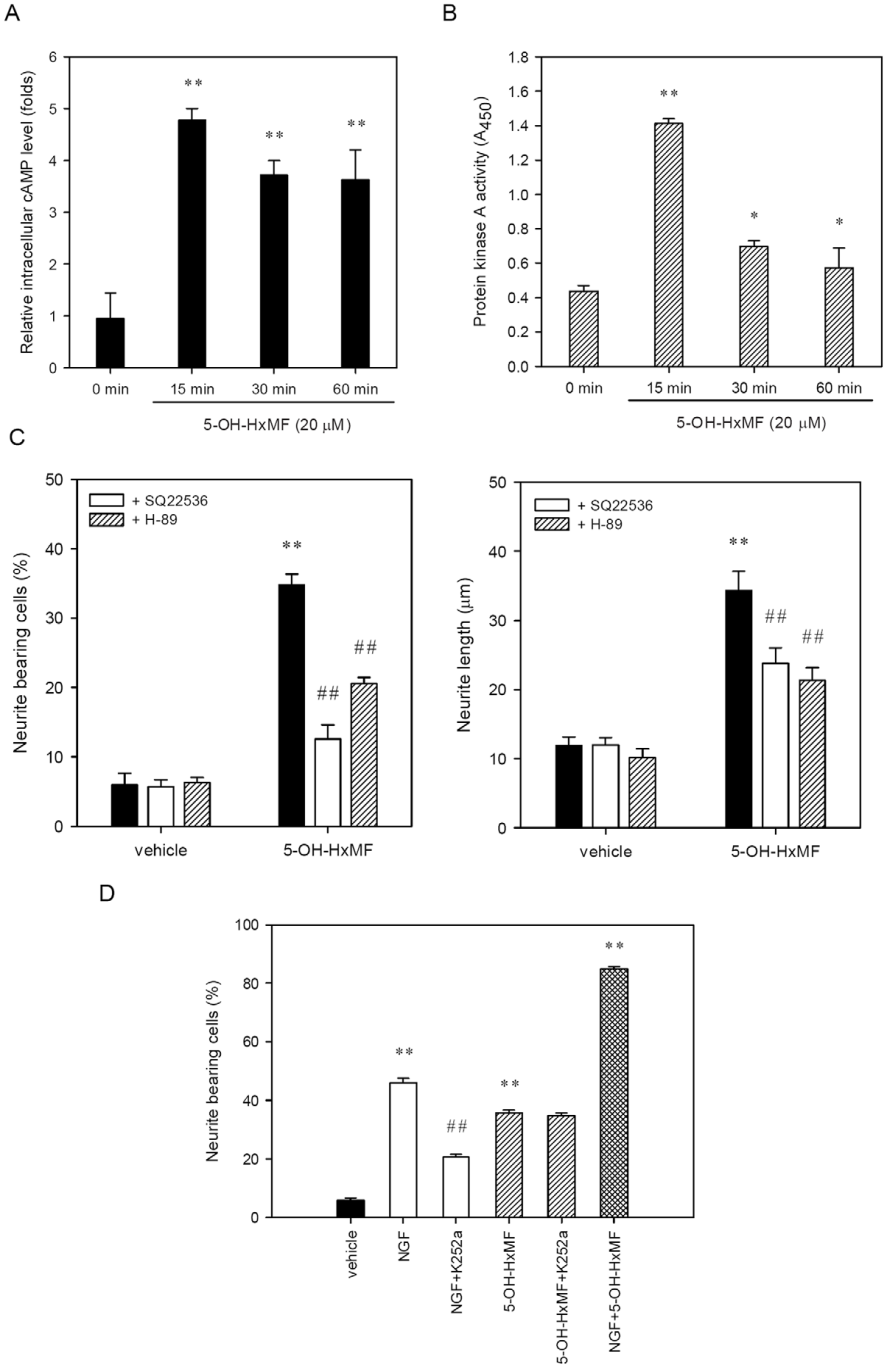
The involvement of cAMP production and protein kinase A (PKA) activation in 5-OH-HxMF-induced neruite outgrowth. PC12 cells were seeded on poly-L-lysine-coated 6-well and then shifted to low serum medium (1% HS and 0.5% FBS) for further 24 h culture. **(A)** Cells were then incubated with 5-OH-HxMF (20 µM) for indicated period and cAMP level was determined by using enzyme immunoassay kit as described in Materials and Methods. **(B)** Cells were then incubated with 5-OH-HxMF (20 µM) for indicated period and PKA activity was detected using ELISA kit as described in Materials and Methods. Data represent the mean ± SD of three independent experiments. *p<0.05 and ** *p*<0.01 represents significant differences compared with 0 min group. **(C)** Cells were then treated for 30 min with inhibitors 500 µM SQ22536 (adenylate cyclase), 10 µM H-89 (PKA) or vehicle (0.1% DMSO), prior to exposure to 5-OH-HxMF (20 µM) for 48 h. Percentage of neurite bearing cells and maximal neurite length for each of the differentiated cells was analyzed as described in Materials and Methods. **(D)** Cells were then treated for 30 min with TrkA antagonist K252a (100 nM) prior to exposure to NGF (100 ng/ml; 0.76 nM) or 5-OH-HxMF (20 µM) for 48 h. The neurite bearing cells were analyzed as described in Materials and Methods. Data represent the mean ± SD from three independent experiments. ** *p*<0.01 represents significant differences compared with vehicle-treated cells. ## *p*<0.01 represents significant differences compared with respective inhibitor-untreated group.

The effect of 5-OH-HxMF on intracellular cAMP levels was further measured using the Enzyme Immunoassay kit as described in Materials and Methods. **Figure 7A** shows that intracellular cAMP levels peaked at 15 min treatment and then slightly decreased until 60 min (*p*<0.01). Moreover, we analyzed the effect of 5-OH-HxMF on the protein kinase A activity using the ELISA-based PKA activity assay kit as described in Materials and Methods and found similar kinetics with peak at 15 min (*p*<0.01) and then fell off (*p*<0.05) (**Figure 7B**). These results indicate the possible involvement of cAMP-dependent PKA signaling pathway in 5-OH-HxMF-mediated CREB activation. The cAMP production and PKA activity may play an essential role for channeling 5-OH-HxMF signaling to CREB activation in PC12 cells.

To determine the role of cAMP-dependent PKA signaling pathway on 5-OH-HxMF-induced neurite outgrowth, we treated cells with 5-OH-HxMF in the presence of specific inhibitors. As shown in **Figure 7C**, exposure of cells to SQ22536 and H-89 significantly attenuated the percentage of neurite-bearing cells from the original 37.3±0.3% to 12.6±2.0% and 20.6±0.9%, respectively (*p*<0.01). The maximal neurite length in those neurite-bearing cells also reduced from the original 37.3±2.8 µm to 23.8±2.3 µm and 21.3±1.8 µm, respectively (*p*<0.01). These results further confirm the vital role of cAMP/PKA/CREB pathway in 5-OH-HxMF-induced neurite outgrowth in PC12 cells. However, the inhibitory potencies of these two inhibitors, especially H-89, on 5-OH-HxMF-induced neurite outgrowth are weaker than that of KG-501 (**Figure 5**), indicating there remains an unidentified CREB upstream signaling.

### 5-OH-HxMF promotes neurite outgrowth in PC12 cells is not via TrkA-mediated actions

It is known that NGF could act on tyrosine kinase receptor A (TrkA) and regulate neuronal differentiation through CREB by causing an elevation of the cAMP, which activates protein kinase A [43]. To evaluate whether 5-OH-HxMF serves as a small-molecular agonist for TrkA, PC12 cells were treated with 20 µM 5-OH-HxMF or 100 ng/ml NGF (as a control) alone or in combination with specific TrkA antagonist K252a (100 nM) for 48 h to analyze the percentage of neurite bearing cells. As shown in **Figure 7D**, K252a significantly inhibited NGF- but not 5-OH-HxMF-induced neurite outgrowth in PC12 cells. In addition, we also found that 5-OH-HxMF represented additive effect on NGF-induced neurite outgrowth. These data suggest that 5-OH-HxMF induces neurite outgrowth by activating a cAMP/PKA-dependent and TrkA-independent signaling pathway coupling with CRE mediated gene transcription.

We further investigated whether the other PKA-independent pathways, such as ERK, PI3-K/Akt, CaMKII, and PKC, also contribute to 5-OH-HxMF-mediated neurite outgrowth in PC12 cells. **Figure S3** shows that 5-OH-HxMF-mediated neurite outgrowth was partly reduced by BIM but not by LY294002, KN-62 (CaMKII) or U0126, which could completely abolish basal and 5-OH-HxMF-induced ERK phosphorylation (**Figure S4**). These results suggest that PKC pathway, but not ERK, PI3K/AKT, or CaMKII, may be associated with the CRE-independent signaling, which also responsible in part for 5-OH-HxMF-mediated neurite outgrowth.

## Discussion

**Figure 8:**
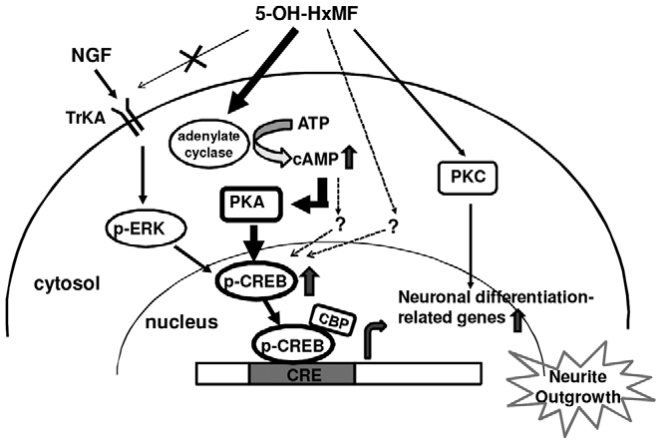
Hypothetic mechanism of 5-OH-HxMF in promoting neurite outgrowth in PC12 cells. 5-OH-HxMF could induce neurite outgrowth associated with expression of neuronal differentiation marker (Figure 2 and 3). 5-OH-HxMF induced neurite outgrowth through stimulating CREB phosphorylation and activation of CREdependent transcription activity (Figure 4 and 5). 5-OH-HxMF stimulated CREB phosphorylation and neurite outgrowth mainly through activation of cAMP-dependent protein kinase A (PKA) and TrkAindependent pathway in PC12 cells (Figure 6 and 7). Another PKAindependent pathway, protein kinase C (PKC), was also partly involved in the 5-OH-HxMF-induced neurite outgrowth. In addition, the PKAindependent but CRE-dependent pathways may also partially contribute to the 5-OH-HxMF-mediated neurite outgrowth. As a result, 5-OHHxMF promotes neurite outgrowth mainly through activation of cAMP/PKA/CREB pathway in PC12 cells.

Therapeutic strategy to stimulate neuronal cell events including proliferation, migration, differentiation, neurite outgrowth, and synatogenesis are needed for several neurodegenerative disorders. Small molecules, such as dietary flavonoids, may work as therapeutic agents that possessed the high neurotrophic potency and involved in numerous effects within the brain [44]. Traditionally, the neuroprotective effects of flavonoids have been attributed to their ability to exert antioxidant actions [45], through their ability to scavenge reactive species, or through their possible influences on intracellular redox status [46]. Recently, it becomes evident that flavonoids are able to stimulate neuronal regeneration and induce neurogenesis via their interactions with critical neuronal intracellular signaling pathways pivotal in controlling neuronal survival and differentiation [47]–[49]. 5-Hydroxy-3,6,7,8,3′,4′- hexamethoxyflavone (5-OH-HxMF) is one of the most abundant hydroxylated PMFs in the sweet orange (*C. sinensis* L.) peel oil [23]. Its known biological functions are limited to anti-inflammatory and anti-cancer activities. For example, it exhibited much stronger inhibitory effects on the growth of the colon cancer cells than its permethoxylated counterpart, 3,5,6,7,8,3′,4′-heptamethoxylflavone [50]; it also induced HL-60 apoptosis [51], [52] and inhibited TPA-induced skin inflammation and tumor promotion [53]. The major findings of this study are that 5-OH-HxMF, can induce PC12 neurite outgrowth accompanied with the expression of neuronal protein GAP-43. In addition, it was demonstrated that 5-OH-HxMF induced neurite outgrowth mainly via increases of intracellular cAMP levels and PKA activity, which further enhanced CREB phosphorylation and CRE dependent transcription in PC12 cells (**Figure 8**).

In PC12 cells, neurite outgrowth can be stimulated by diverse growth factors activating corresponding receptors: NGF, BDGF, fibroblast growth factor (FGF), and epidermal growth factor (EGF) accompanied by more than a 10-fold increase of GAP-43 and its mRNA levels [35], [54], [55]. GAP-43 is one of the abundant nerve ending proteins with the function of taking an incoming signal and transducing it to the effectors and is therefore regarded as ‘‘signal’’ protein”. It plays a critical role to regulate nerve sprouting and the actin cytoskeleton [56]. In this research, we found that 5-OH-HxMF and NGF stimulated neurite outgrowth with significantly higher expression of GAP-43 mRNA and protein after 24 h incubation.

It has been suggested that phosphorylation and dephosphorylation of CREB control its ability to regulate transcription which is associated with neuronal function such as synapse re-modeling, increases in neuronal spine density and synaptic plasticity [49]. In this research, we clearly detected 5-OH-HxMF treatment induced CREB phosphorylation, CRE-dependent transcription activity and CREB target gene c-fos mRNA expression in PC12 cells. Addition of CREB:CBP antagonist, KG501, suppressed 5-OH-HxMF-induced luciferase activity in pCRE-Luc transfected PC12 cells and neurite outgrowth. Our findings indicate the close correlation between CREB activation and the neuritogenesis in 5-OH-HxMF-treated PC12 cells. Previously, Nagase et al. compared the neurotrophic potencies of six citrus flavonoids, namely nobiletin, 5-demethylnobiletin, tangeretin, sinensetin, 6-demethoxytangeretin and 6-demethoxynobiletin, in PC12D cells, a derivative of the PC12 cell line which extends neurites very rapidly in response to NGF, even when RNA synthesis is blocked. They found that among the test compounds, nobiletin most potently enhanced CREB phosphorylation, CRE-dependent transcription and neurite outgrowth [28]. Herein, we found that 5-OH-HxMF exhibited similar stimulatory activity as nobiletin for neurite outgrowth and CREB phosphorylation. It is known that NGF-responsive CREB activation also involved in the neuronal survival and neuroprotection. Thus, we further examined the neuroprotective effect of 5-OH-HxMF and nobiletin against serum deprivation-induced cell death. We found that nobiletin but not 5-OH-HxMF significantly increased the cell viability in serum free cultured PC12 cells (data not shown). This result implies that 5-OH-HxMF induced CREB activation through PKA involved in neuritogenic action, but may be not play a vital role in the cytoprotective effect against serum deprivation induced cell apoptosis.

The activation of various signaling pathways, including cAMP-dependent protein kinase A (PKA), PI3 k/Akt, protein kinase C (PKC), calcium-calmodulin kinase II/IV (CaMKII/IV) and ERK have been linked with the control of de novo protein synthesis in the context of LTP (long term, synaptic plasticity and memory) and converge to signal to CREB [14]. In this study, we found that the adenylate cyclase inhibitor SQ22536 and the PKA inhibitor H-89 significantly blocked the potentiation of 5-OH-HxMF-induced neurite outgrowth, CRE transcription activity and CREB phosphorylation. Moreover, we found that the MEK inhibitor U0126, the PKC inhibitor BIM, the PI3K inhibitor LY294002 and the CaMKII inhibitor KN-62 did not significantly change 5-OH-HxMF-induced CRE transcription activity. Additionally, we found that 5-OH-HxMF induced the accumulation of intracellular cAMP and PKA activity. The role how 5-OH-HxMF affects the intracellular cAMP level remains unclear. Whether it is through activation of adenylate cylase activity alone or in combination with inhibition of phosphodiesterases (PDEs), which catalyze the hydrolysis of cAMP and thereby increase intracellular cAMP concentration to activate PKA, is an open question.

Cross talk has been found existing between cAMP/PKA and ERK signaling pathways in PC12 cells [57]. It has been found that nobiletin induces neurite outgrowth by activating a cAMP/PKA/MEK/Erk/MAP kinase-dependent and TrkA-independent signaling pathway coupling with CRE mediated gene transcription in PC12 D cells [27]. The metabolite of nobiletin, 4′-demethylnobiletin was shown to stimulate the phosphorylation of ERK and CREB and enhances CRE-mediated transcription by activating a PKA/MEK/ERK pathway in cultured hippocampal neurons and rescues learning impairment associated with NMDA receptor antagonism [33]. In this study, we found that 5-OH-HxMF increased the cellular levels of cAMP and PKA activity. Addition of SQ22536, a adenylate cyclase inhibitor; and H-89, a PKA inhibitor, significantly blocked the potentiation of 5-OH-HxMF-induced neurite outgrowth, CREB phosphorylation and CRE transcription activity. On the other hand, K252a, a TrkA antagonist, did not affect 5-OH-HxMF-induced neurite outgrowth. While cells co-treated with NGF and 5-OH-HxMF showed additive effect on neurite outgrowth. Our findings indicate 5-OH-HxMF induced PC12 cell neurite outgrowth through cAMP/PKA/CREB pathway which is not associated with TrKA activation.

In this study, we found that while PC12 cells treated with inhibitor H-89 only partly reversed 5-OH-HxMF-induced neurite outgrowth (**Figure 7C**); however, inhibition of CREB activation by KG-501 almost completely blocked the induction (**Figure 5C**). Thus, a PKA-independent but CRE-dependent pathway may be involved in 5-OH-HxMF-mediated neurite outgrowth. We next examined the role of PKA-independent pathways in 5-OH-HxMF-induced neurite outgrowth by specific kinase inhibitors. Inhibition of PKC, but not MEK1/2, PI3-K/Akt, or CaMKII, reduced the neurite outgrowth. PKC inhibitor modestly decreased the percentage of 5-OH-HxMF-induced neurite outgrowth by approximately 10%. However, inhibition of PKC did not significantly affect CRE-mediated transcription activity (**Figure 6A**). This finding indicates PKC pathway might be partly involved in the 5-OH-HxMF-mediated neuritogenic action.

It has been reported that certain cAMP-dependent activities were not inhibited by PKA inhibitor [58]. In this study, we found that SQ22536 was more effective than H-89 at reducing 5-OH-HxMF-induced neurite outgrowth. This result implies that a cAMP-mediated but PKA-independent pathway may also partially contribute to the 5-OH-HxMF-mediated CREB activation and neuritogenic action. Moreover, our data showed that 5-OH-HxMF could transiently activate ERK which could be abolished by U0126, an ERK phosphorylation inhibitor. However, 5-OH-HxMF-induced CREB activation or neurite outgrowth was not significantly affected by U0126. Therefore, there is no crosstalk between ERK activation and 5-OH-HxMF-induced cAMP/PKA pathway, CREB activation, or neuronal differentiation. Taken together, these findings suggest that cAMP/PKA-dependent, but not TrkA- or ERK-dependent, signaling pathway coupling with CRE mediated gene transcription is involved in the mechanisms of 5-OH-HxMF-induced neurite outgrowth.

## Materials and Methods

### Chemicals

5-Hydroxy-3,6,7,8,3′,4′-hexamethoxyflavone and nobiletin were purified as described before [23]. Poly-L-lysine, dimethyl sulfoxide (DMSO), KN-62 [1-[N,O-bis(5-isoquinolinesulfonyl)-N-methyl-L-tyrosyl]-4- phenylpiperazine], H-89 [N-[2-((*p*-Bromocinnamyl)amino)ethyl]-5-isoquinolinesulfonamide], 2-naphthol AS-E phosphate (KG-501), forskolin as well as other chemicals were purchased from Sigma-Aldrich Co. (St. Louis, MO) unless otherwise indicated. LY294200 [2-(4- morpholinyl)-8-phenyl-4H -1-benzopyran-4-one], a PI3-K inhibitor, and U0126 [1,4-diamino-2,3-dicyano-1,4- bis (2-aminophenylthio)butadiene], a selective and potent inhibitor of MEK activity and activation of ERK1/2, were purchased from Promega (Madison, WI, USA). Bisindolylmaleimide I, a protein kinase C inhibitor, was purchase from Cayman chemical (Ann Arbor, MI, USA). SQ22536 [9-(Tetrehydro-2-furyl) adenine], a cell-permeable adenylate cyclase inhibitor, and TrkA antagonist K252a were purchased from Enzo Life sciences (Ann Arbor, MI, USA). The mouse 7S nerve growth factor (NGF) was purchased from Millipore (Billerica, MA, USA).

### Cell culture

PC12 cell, the rat adrenal pheochromocytoma cell line, was obtained from Bioresource Collection and Research Center (Hsinchu, Taiwan) and maintained in complete medium which contains RPMI-1640 (Sigma-Aldrich), 2 mM glutamine, 1.5 g/L sodium bicarbonate, 4.5 g/L glucose, 10 mM HEPES and 1 mM sodium pyruvate, supplemented with 10% heat-inactivated horse serum (HS) (Invitrogen, Carlsbad, CA, USA) and 5% fetal bovine serum (FBS)(Biological Industries, Kibbutz Haemek, Israel) in 5% CO2 incubator at 37°C.

### Analysis of PC12 cell numbers by MTT assay

The survival cell numbers were measured by the mitochondrial-dependent reduction of 3-(4, 5-dimethylthiazol-2-yl)-2, 5-diphenyl tetrazolium bromide (MTT) to purple formazan. Briefly, PC12 cells were incubated with MTT solution (1 mg/ml final concentration) for 4 h at 37°C followed by centrifugation at 8,000×g for 4 min. The medium was carefully removed by aspiration, then the formazan crystals were dissolved in dimethyl sulfoxide (DMSO). The extent of the reduction of MTT was determined by measurement of the absorbance at 550 nm.

### Analysis of neurite outgrowth of PC12 cells

Morphological analysis and quantification of neurite bearing cells were carried out using phase-contrast microscope as described previously [59], [60]. Briefly, PC12 cells (3×10^5^/ml) were seeded on poly-L-lysine-coated 6-well plates in the normal serum medium for 24 h. The RPMI medium containing low serum (1% HS and 0.5% FBS) was replaced prior to exposure to vehicle (0.1% DMSO) or indicated reagents. After an additional 48 h of incubation, neurite outgrowth of PC12 cell was observed under an inverted microscope (Olympus IX71) using phase-contrast objectives and photographed by the digital camera. At least 100 cells in each of ten randomly separated fields were scored and the proportion of cells with neurites greater than or equal to the length of one cell body were scored positive for neurite outgrowth, and expressed as a percentage of the total cell number in ten fields. The neurite extension length was also measured for all identified positive neurite-bearing cells in a field by tracing the longest length of neurite per cell using Image J software (NIH Image software). The value of neurite length (average maximal neurite length per neurite-bearing cell in ten fields) was calculated and data from the ten fields in each well was designated as one experiment. Experiments were repeated at least three times on separate days and data are expressed as mean ±SD.

For detection of neurite outgrowth by indirect immunofluorescence, PC12 cells were seeded on poly-L-lysine-coated coverslip in the normal serum medium for 24 h, then the RPMI medium containing low serum medium was replaced prior to exposure to NGF. After an additional 48 h of incubation, PC12 cells were fixed with 3.7% formaldehyde in phosphate-buffered saline (PBS) for 15 min, washed, permeabilized with 0.1% Triton X-100, and soaked in blocking buffer (PBS containing 1% BSA) for 60 min at room temperature. After blocking, immunostaining was conducted by incubation with the primary anti-GAP-43 antibody (Millipore, Billerica, MA, USA), washed, and incubated the secondary Alexa Fluor 488-conjugated goat anti-mouse IgG antibody (Invitrogen, Carlsbad, CA, USA) for 60 min. The cells were then washed four times in PBS and incubated with 4′,6- diamidino-2-phenylindole (DAPI) solution for 2 min for nuclear staining. The coverslips were again washed in PBS, drained, and mounted with Fluoromount G (Andes import). The cells were viewed and photographed on the inverted fluorescence microscope (Olympus IX71).

### Reverse transcription quantitative PCR (RT-Q-PCR) analysis of GAP-43 and c-fos

PC12 cells (1×10^6^/ml) were seeded on poly-L-lysine-coated 6-well plates in normal medium for 24 h. The cells were then shifted to low serum (1% HS and 0.5% FBS) as indicated for 24 h prior to exposure to vehicle (0.1% DMSO) or indicated reagents for indicated period. Total cellular RNA was prepared using Total RNA mini Kit (Geneaid, Taipei, Taiwan). Reverse transcription of 2 µg RNA was performed using High Capacity cDNA reverse transcription kit (Applied Biosystems, Foster City, CA, USA). Quantitative real-time PCR was performed with 2 µL cDNA obtained above in 25 µL containing 200 nM primers [GAP-43, 5′-CTAAGGAAAGTGCCCGACAG-3′ (forward) and 5′-GCAGGAGAGACAGGGTTCAG-3′ (reverse); β-actin, 5′-CCTCTGAACCCTAAGGCCAA-3′ (forward) and 5′-AGCCTGGATGGCTACGTACA-3′ (reverse)[59]; c-fos, 5′-TCTCCTGAAGAGGAAGAGAAACGG-3′ (forward) and 5′-TCTGCAACGCAGACTTCTCG-3′ (reverse) [61]] and Maxima SYBR Green/ROX qPCR Master Mix (Fermentas, Burlington, CA). Amplification was conducted in an ABI Prism 7300 Real-Time PCR System. PCR conditions were as follows: 94°C for 4 min, 40 cycles at 94°C for 1 min, 58°C for 1 min, and 72°C for 1 min. The ΔΔC_t_ method was used for data analysis of GAP-43 mRNA expression estimated in triplicate samples and normalized to β-actin expression levels.

### Western blotting analysis of GAP-43, CREB, and ERK proteins

PC12 cells (1×10^6^/ml) were seeded on poly-L-lysine-coated 100 mm dishes in normal serum medium for 24 h, then shifted to low serum (1% HS and 0.5% FBS) as indicated for 24 h prior to exposure to vehicle (0.1% DMSO) or indicated reagent for indicated periods. Cells were washed with PBS, scraped in ice cold RIPA buffer (Thermo Fisher Scientific, Inc., Rockford, IL) and incubated on ice for 15 min. The cellular debris was removed by centrifugation (8,000×g for 15 min) at 4°C and the cell lysate was carefully transferred to the microcentrifuge tube. The protein concentration was measured by the Bradford method (Bio-Rad Laboratories, Hercules, CA, USA) using bovine serum albumin as a standard.

Cell lysate (30 µg) was separated on 10% SDS-PAGE and transferred onto PVDF membrane (PerkinElmer, Boston, MA, USA) at 25 volt overnight at 4°C. The membranes were blocked at 4°C in PBST blocking buffer (1% non-fat dried milk in PBS containing 0.1% Tween-20) for 8 h. Blots were incubated with the appropriate antibodies overnight at 4°C: anti-GAP-43 (1∶1000) (Millipore, Billerica, MA, USA), anti-β-actin (1∶8000) (Sigma-Aldrich), anti-phospho-CREB(Ser-133) (1∶1000), anti-CREB (1∶1000), anti-ERK1/2 (1∶1000) and anti-phospho-ERK1/2 (1∶1000) (Cell Signaling Technology, Inc.). After three washes with PBST, the blots were incubated with appropriate horseradish peroxidase-conjugated secondary antibodies (1∶10,000) for 1 h. The blots were washed again and detected the proteins of interest by Western Lightning^TM^ Chemiluminescence Reagent *Plus* (PerkinElmer, Boston, MA, USA) according to the manufacturer's instructions, and then the chemiluminescence signal was visualized with X-ray film.

### Reporter gene assay of cyclic AMP response element (CRE)-mediated transcription activity

PC12 cells (2×10^5^/well) were seeded on poly-L-lysine-coated 24-wells plate in DMEM containing 10% HS and 5% FBS medium for 24 h. For transient transfection, cells were cotransfected with the pCRE-Luc Cis-reporter plasmid (Stratagene, La Jolla, CA, USA) and *Renilla* luciferase vector (Promega) using Lipofectamine 2000 (Invitrogen). Twenty-four hours after transfection, cells were treated with vehicle (0.1% DMSO), 5-OH-HxMF (20 µM), or nobiletin for 8 h and harvested by using Passive Lysis Buffer (Promega). For treatment of PC12 cells with inhibitors, the transfected cells were pre-incubated with different inhibitors for 30 min, then treated with indicated agents for 8 h before harvesting the cell lysates. Luciferase activities were determined by the Dual-Luciferase Reporter Assay System Kit (Promega) according to the manufacturer's instructions. The intensity of the luciferase reactions measured in the lysates of the transfectants was normalized to their *Renilla* luciferase activity, which was used as an internal control.

### Analysis of cyclic AMP (cAMP) levels

PC12 cells (1×10^6^/ml) were seeded on poly-L-lysine-coated 6-well plates in normal medium for 24 h. The cells were then shifted to low serum (1% HS and 0.5% FBS) as indicated for 24 h prior to exposure to 5-OH-HxMF compound for indicated periods. Cells were treated with 0.1 M HCl after removing the culture media and incubated for 10 min to verify cell lysis. The cell lysates were centrifuged at room temperate and the supernatant was used directly in the assay. The intracellular cyclic AMP level was measured by the Direct Cyclic AMP Enzyme Immunoassay Kit (Enzo Life Sciences) according to the manufacturer's instructions.

### Analysis of protein kinase A (PKA) activity

A nonradioactive protein kinase A (PKA) activity assay kit (Enzo Life Sciences) was used to measure PKA activity in the samples. PC12 cells (1×10^6^/ml) were seeded on poly-L-lysine-coated 100 mm dishes in normal serum medium for 24 h, then shifted to low serum (1% HS and 0.5% FBS) as indicated for 24 h prior to exposure to 5-OH-HxMF compound for indicated periods. Cellular proteins were collected using lysis buffer according to the manufacturer's instruction. PKA substrate microtiter plate, which was pre-coated with PKA substrate, was soaked with kinase assay dilution buffer for 10 min at room temperature. 30 µL of cell lysates (100 ng) or PKA standard (10 ng) were then added, followed by the addition of ATP to initiate the reaction. After incubation at 30°C for 90 min, the reaction mixture was removed from the plate, and phosphospecific substrate antibody was added to each well and incubated at room temperature for 60 min. The liquid was aspirated and wells were repeatedly washed. HRP-conjugated secondary anti-rabbit IgG was then added to each well and incubated for another 30 min at room temperature. The wash was repeated after incubation and TMB substrate solution was added to each well. Stop solution was added after 30–60 min and the 96-well plate was read at 450 nm in a microplate reader.

### Statistical Analysis

All experiments were repeated at least three times. The results were analyzed by Student's unpaired *t*-test and a *p* value of <0.05 was taken to be significant.

## Supporting Information

Figure S1 **Effects of 5-OH-HxMF and nobiletin on the cell viability of PC12 cells.** PC12 cells (1×10^5^/well) were seeded on 24-well plates in normal serum (10% HS and 5% FBS), low serum medium (1% HS and 0.5% FBS) and exposed to vehicle (0.1% DMSO), NGF (as a positive control), 5-OH-HxMF or nobiletin (0–50 µM) for 48 h. The relative cell counts were determined by MTT assay as described in the Materials and Methods and expressed as percentage of control group, which represents the cell counts prior to medium change. Data represent the mean ± SD from three independent experiments. *p<0.05 and **p<0.01 represent significant differences compared with control group cells.
(TIF)

Figure S2 **Analysis of neurite outgrowth in PC12 cells.** PC12 cells were seeded on poly-L-lysine-coated 6-well plates in low serum medium for 24 h prior to exposure to vehicle (0.1% DMSO) or NGF for additional 48 h. Cell morphology was observed using phase-contrast microscopy and photographed by the digital camera. **(A)** Phase contrast micrographs of PC12 cells. Arrowheads indicate the neurite bearing cells in vehicle- or NGF (100 ng/ml)-treated groups. Scale bar, 100 µm. **(B)** PC12 cells were treated with NGF (indicated concentration) for 48 h or 96 h. Neurite bearing cells were analyzed as described in Materials and Methods. Data represent the mean ± SD from three independent experiments. *p<0.05 and **p<0.01 represents significant differences compared with those of the vehicle-treated cells. **(C)** PC12 cells were seeded on poly-L-lysine-coated coverslip and cultured in the low serum medium for NGF (100 ng/ml) treatment for 48 h. Indirect immunofluorescence assay for detecting GAP-43 protein as described in Materials and Methods. GAP-43 protein was detected by immunofluorescence microscope (green). DAPI stains nuclei (blue). Arrowheads indicate the neurite bearing cells. Scale bar; 100 µm.
(TIF)

Figure S3 **Effects of protein kinase inhibitors on the 5-OH-HxMF-induced neurite outgrowth.** PC12 cells were seeded on poly-L-lysine-coated 6-well plates in normal serum medium for 24 h. Cells were then shifted to low serum medium (1% HS and 0.5% FBS) for 24 h and then were pre-treated for 30 min with inhibitors 10 µM U0126 (MEK1/2), 2.5 µM BIM (PKC), 40 µM LY294002 (PI3-K/Akt), and 10 µM KN-62 (CaMKII), respectively, followed by exposure to 5-OH-HxMF (20 µM) for 48 h. Neurite bearing cells were analyzed as described in Materials and Methods. Data represent the mean ± SD from three independent experiments. ** *p*<0.01 represents significant differences compared with vehicle-treated cells. ## *p*<0.01 represents significant differences compared with respective inhibitor-untreated group.
(TIF)

Figure S4 **Effects of 5-OH-HxMF on the phosphorylation of ERK proteins.** PC12 cells were seeded on poly-L-lysine-coated 100 mm dishes in normal medium for 24 h and then shifted to low serum medium (1% HS and 0.5% FBS) for 24 h prior to exposure to indicated agents. Cells were treated with 5-OH-HxMF (20 µM) for 0 min, 15 min, and 30 min. Phosphor-ERK1/2 (p-ERK1/2) and total ERK1/2 proteins were analyzed by Western blotting as described in Materials and Methods. The immunoblot experiments were replicated at least three times and a representative blot was shown.
(TIF)
