# Supplementary figures and images for: Neurotrophic Effect of Citrus 5-Hydroxy-3,6,7,8,3′,4′-Hexamethoxyflavone: Promotion of Neurite Outgrowth via cAMP/PKA/CREB Pathway in PC12 Cells
Source: PLoS One. 2011 Nov 29;6(11):e28280. doi: 10.1371/journal.pone.0028280 (PMC3226691; doi:10.1371/journal.pone.0028280)

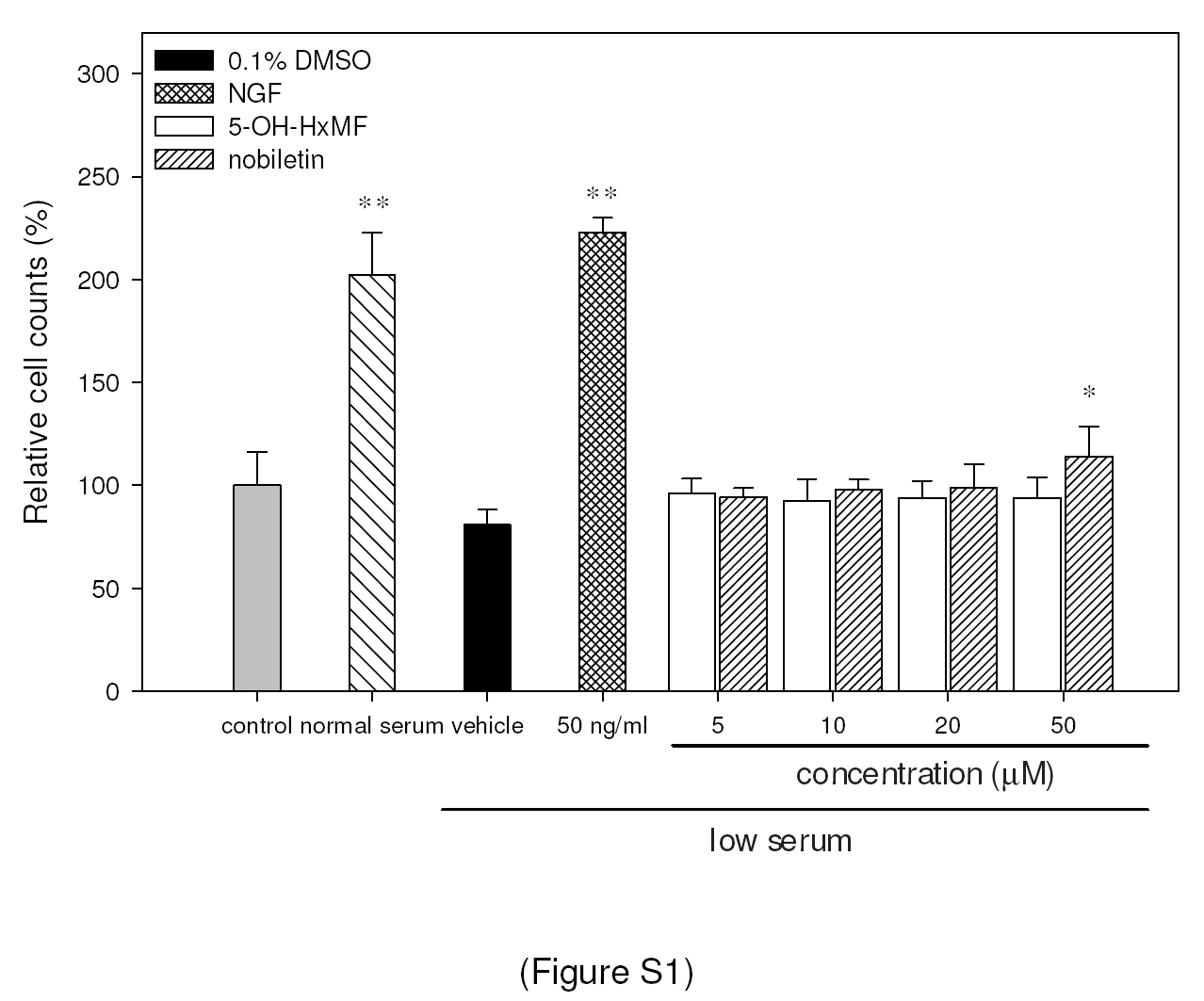

Supplement: Figure S1 — Effects of 5-OH-HxMF and nobiletin on the cell viability of PC12 cells. PC12 cells (1×10^5^/well) were seeded on 24-well plates in normal serum (10% HS and 5% FBS), low serum medium (1% HS and 0.5% FBS) and exposed to vehicle (0.1% DMSO), NGF (as a positive control), 5-OH-HxMF or nobiletin (0–50 µM) for 48 h. The relative cell counts were determined by MTT assay as described in the Materials and Methods and expressed as percentage of control group, which represents the cell counts prior to medium change. Data represent the mean ± SD from three independent experiments. *p<0.05 and **p<0.01 represent significant differences compared with control group cells. (TIF) [file pone.0028280.s001.tif]

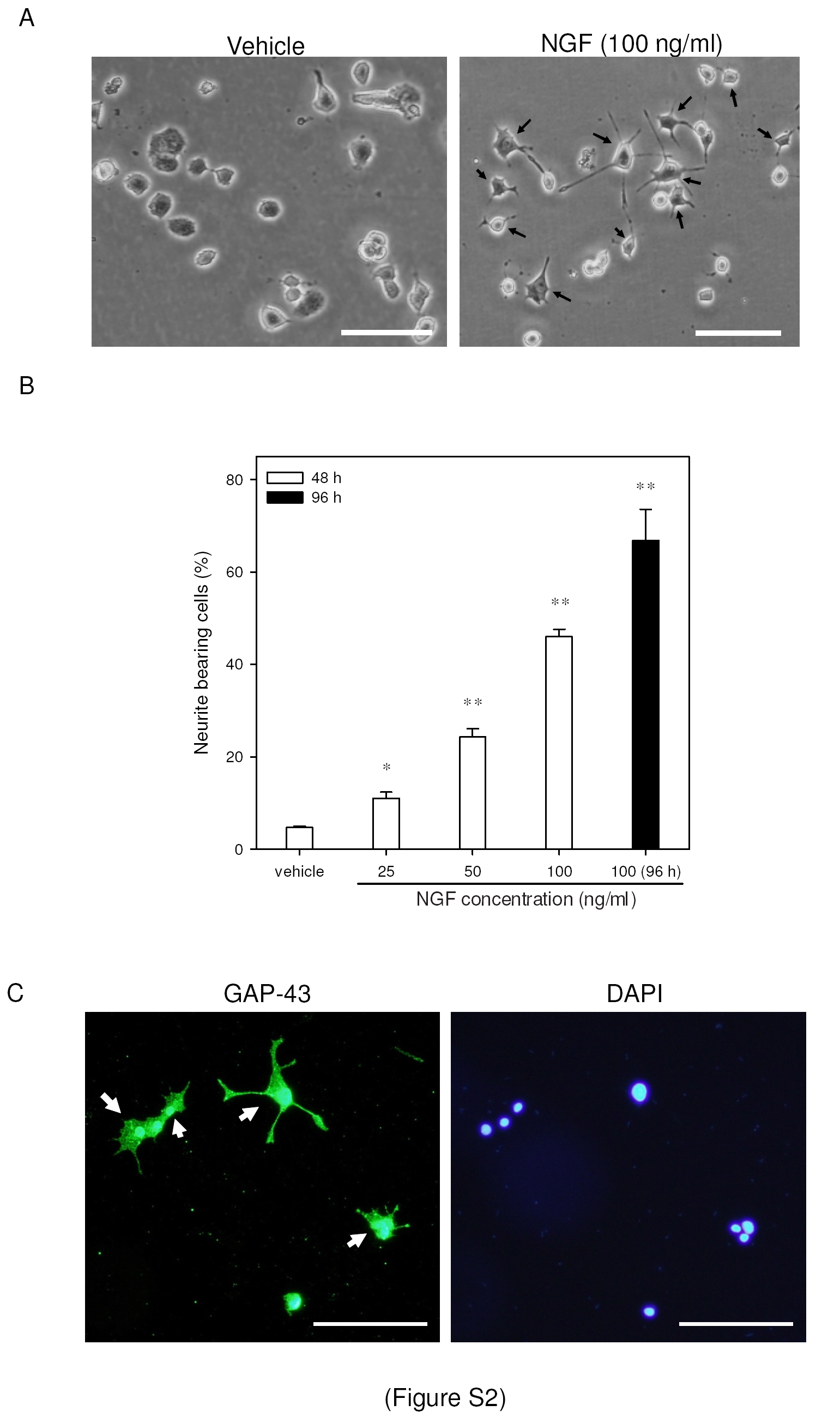

Supplement: Figure S2 — Analysis of neurite outgrowth in PC12 cells. PC12 cells were seeded on poly-L-lysine-coated 6-well plates in low serum medium for 24 h prior to exposure to vehicle (0.1% DMSO) or NGF for additional 48 h. Cell morphology was observed using phase-contrast microscopy and photographed by the digital camera. (A) Phase contrast micrographs of PC12 cells. Arrowheads indicate the neurite bearing cells in vehicle- or NGF (100 ng/ml)-treated groups. Scale bar, 100 µm. (B) PC12 cells were treated with NGF (indicated concentration) for 48 h or 96 h. Neurite bearing cells were analyzed as described in Materials and Methods. Data represent the mean ± SD from three independent experiments. *p<0.05 and **p<0.01 represents significant differences compared with those of the vehicle-treated cells. (C) PC12 cells were seeded on poly-L-lysine-coated coverslip and cultured in the low serum medium for NGF (100 ng/ml) treatment for 48 h. Indirect immunofluorescence assay for detecting GAP-43 protein as described in Materials and Methods. GAP-43 protein was detected by immunofluorescence microscope (green). DAPI stains nuclei (blue). Arrowheads indicate the neurite bearing cells. Scale bar; 100 µm. (TIF) [file pone.0028280.s002.tif]

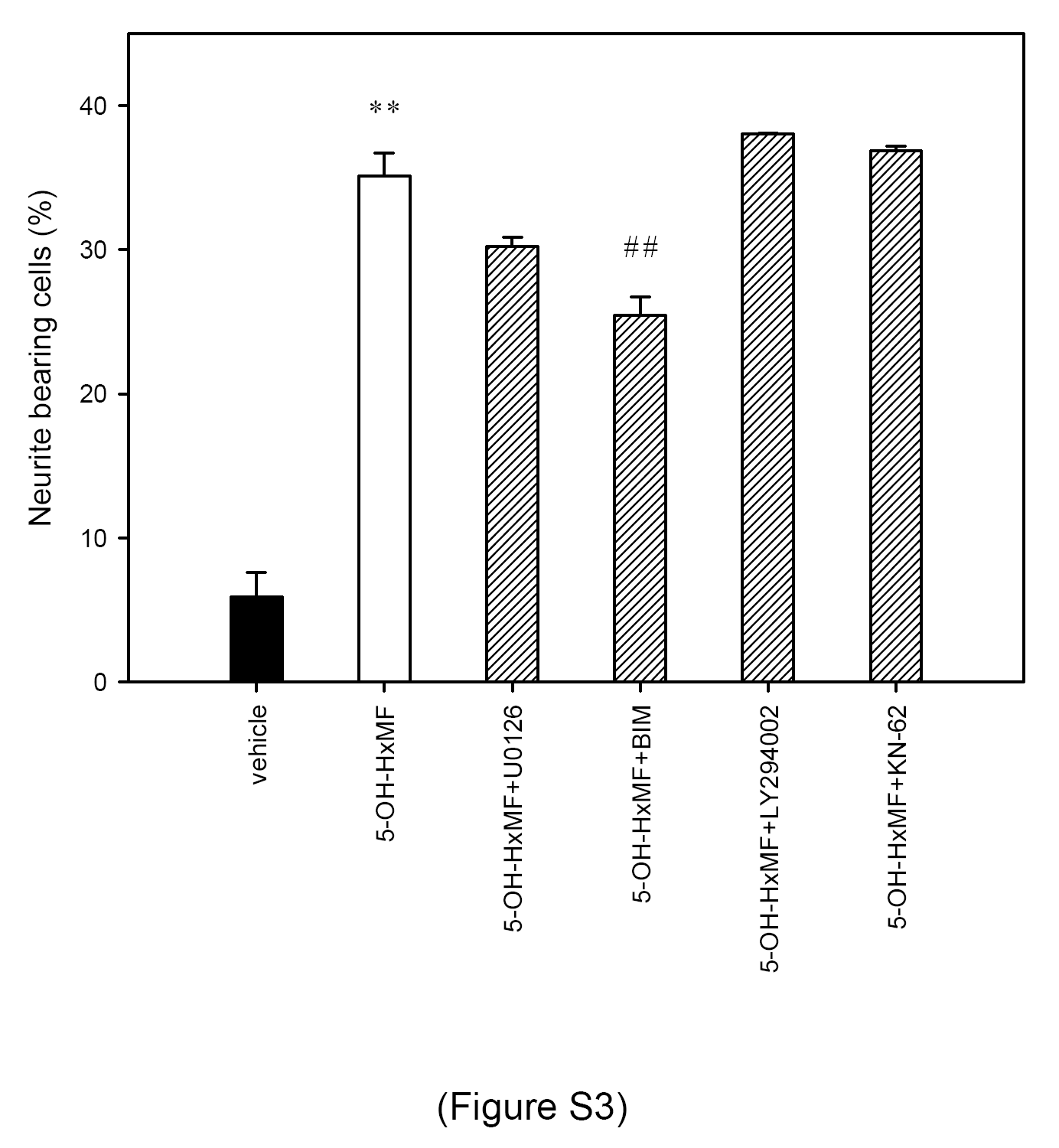

Supplement: Figure S3 — Effects of protein kinase inhibitors on the 5-OH-HxMF-induced neurite outgrowth. PC12 cells were seeded on poly-L-lysine-coated 6-well plates in normal serum medium for 24 h. Cells were then shifted to low serum medium (1% HS and 0.5% FBS) for 24 h and then were pre-treated for 30 min with inhibitors 10 µM U0126 (MEK1/2), 2.5 µM BIM (PKC), 40 µM LY294002 (PI3-K/Akt), and 10 µM KN-62 (CaMKII), respectively, followed by exposure to 5-OH-HxMF (20 µM) for 48 h. Neurite bearing cells were analyzed as described in Materials and Methods. Data represent the mean ± SD from three independent experiments. ** p<0.01 represents significant differences compared with vehicle-treated cells. ## p<0.01 represents significant differences compared with respective inhibitor-untreated group. (TIF) [file pone.0028280.s003.tif]

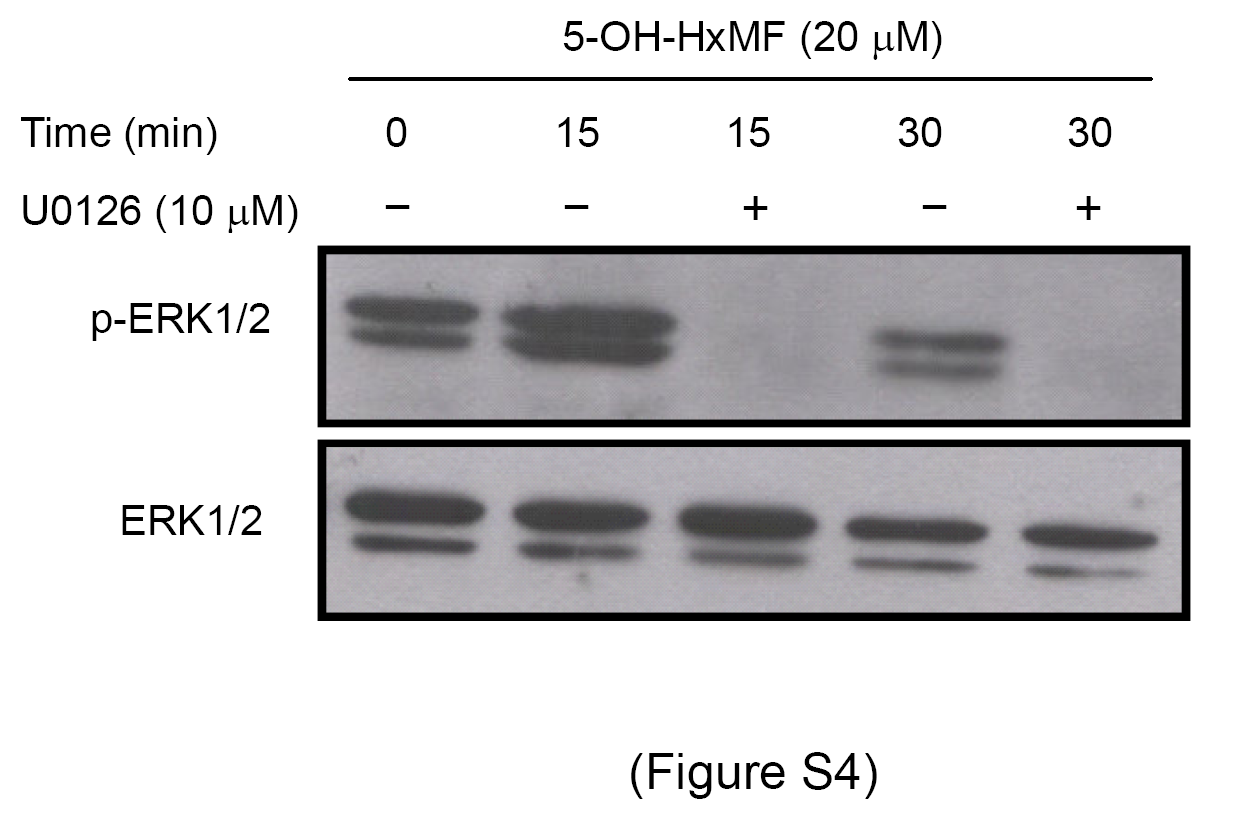

Supplement: Figure S4 — Effects of 5-OH-HxMF on the phosphorylation of ERK proteins. PC12 cells were seeded on poly-L-lysine-coated 100 mm dishes in normal medium for 24 h and then shifted to low serum medium (1% HS and 0.5% FBS) for 24 h prior to exposure to indicated agents. Cells were treated with 5-OH-HxMF (20 µM) for 0 min, 15 min, and 30 min. Phosphor-ERK1/2 (p-ERK1/2) and total ERK1/2 proteins were analyzed by Western blotting as described in Materials and Methods. The immunoblot experiments were replicated at least three times and a representative blot was shown. (TIF) [file pone.0028280.s004.tif]
